# Recurrent Retinal Detachment in Stickler Syndrome

**DOI:** 10.21203/rs.3.rs-3941698/v1

**Published:** 2024-04-05

**Authors:** Timothy Chen, Marjan Fooladi, Michelle Alabek, Hannah Scanga, Kelly Tripi, Ken Nischal, Joseph Martel

**Affiliations:** UPMC Vision Institute; Children’s Hospital of Pittsburgh of UPMC; Children’s Hospital of Pittsburgh of UPMC; Children’s Hospital of Pittsburgh of UPMC; UPMC

## Abstract

**Objective:**

To assess clinical factors leading to recurrent retinal detachment (RD) and characteristics of recurrence in patients with Stickler Syndrome.

**Methods:**

Retrospective case series study of patients with clinical diagnosis of Stickler Syndrome who underwent rhegmatogenous RD repair. Recurrent RD after initial surgery was categorized as “early” if the recurrence was within 1 year or “late” if greater than 1 year.

**Results:**

Thirty eyes from 22 patients underwent rhegmatogenous RD repair. For initial repair, 13 eyes underwent pars plana vitrectomy combined with scleral buckling (PPV/SB), 16 eyes underwent primary scleral buckling (SB), and 1 eye underwent pneumatic retinopexy (PnR). Recurrent RD occurred in 6 (46%) PPV/SB eyes (5 early and 1 late), 10 (63%) SB eyes (3 early and 7 late), and 0 (0%) PnR eyes (p = 0.61). PPV/SB was preferred for eyes presenting with total detachment (82%), giant retinal tears (100%), and proliferative vitreoretinopathy (PVR) (80%). For eyes with early recurrent RD, 6 (75%) developed PVR leading to recurrence. For eyes with late recurrent RD, 7 (87.5%) developed a new retinal break leading to recurrence, including 4 with a break posterior to the buckle indentation apex. At last follow-up, median LogMAR visual acuity was 0.68 for eyes with recurrent RD compared to 0.29 for eyes without recurrence (p = 0.27).

**Conclusions:**

Early recurrent RD was mostly caused by PVR, while late recurrent RD was mostly due to new retinal breaks. Eyes with seemingly uncomplicated rhegmatogenous RD repair with primary SB remained at high risk for late re-detachment.

## Introduction

Stickler Syndrome is the most common inherited cause of rhegmatogenous retinal detachments (RD).^1^ It is a collagen disorder identified by the ocular manifestations of abnormal vitreous development and unusually high myopia, with systemic findings of cranio-facial abnormalities, hearing loss, and arthropathy.^2^ Approximately 40–60% of patients with Stickler Syndrome will develop a rhegmatogenous RD in their lifetime.^3^ Several factors contribute to the complexity of RD repair in patients with Stickler Syndrome, including the presence of multiple retinal tears peripherally and posteriorly, giant retinal tears (GRT), proliferative vitreoretinopathy (PVR), young age at presentation, and an abnormal vitreous with an anomalous vitreoretinal interface. These considerations present a therapeutic challenge to the vitreoretinal surgeon for prophylaxis strategies and for long-term sustained retinal reattachment Retrospective studies have demonstrated that we are able to achieve high levels of anatomic success in Stickler Syndrome-associated RD, though this often requires multiple surgeries due to recurrent RD.^4^ In this study, we aimed to assess factors leading to the development of recurrent RD and characterize features of the eyes at recurrence. Furthermore, because patients often present early in life, we wanted to explore the development of late recurrent RD.

## Methods

We performed a retrospective case series study of patients with a clinical diagnosis of Stickler Syndrome who had undergone surgery for rhegmatogenous RD repair at our institution from 2006–2021. A recurrent RD after initial surgery was categorized as “early” if the detachment recurred within 1 year or “late” if greater than 1 year. When available, molecular diagnosis was noted. The study was approved by our institution’s Institutional Review Board (STUDY 220100600).

Statistical analysis was performed in R and GraphPad Prism (GraphPad Software, Boston, MA). Log-rank test and Mann Whitney U test were used in their corresponding analyses for tests of statistical significance. Odds ratio (OR) were reported with 95% confidence intervals (CI). For conversion to logMAR values, visual acuity of count fingers (CF), hand movements (HM), light perception (LP), and no light perception (NLP) were assigned to values of 2.1, 2.4, 2.7, and 3.0 respectively.^5^

## Results

We identified 30 eyes from 22 patients that underwent rhegmatogenous RD repair (Table 1). The median age at time of surgery was 10 years (range 4 to 40 years old) with 16 (72%) males. Two patients presented with bilateral RD, 1 patient already had an inoperable RD in the fellow eye at presentation, and 6 more patients had eventual bilateral involvement. For those 6 patients, the median time to fellow eye RD was 3.8 years (range 23 days to 10 years). Fourteen (64%) patients had molecular confirmation, with the majority possessing a monoallelic COL2A1 mutation.

**Figure 1:**
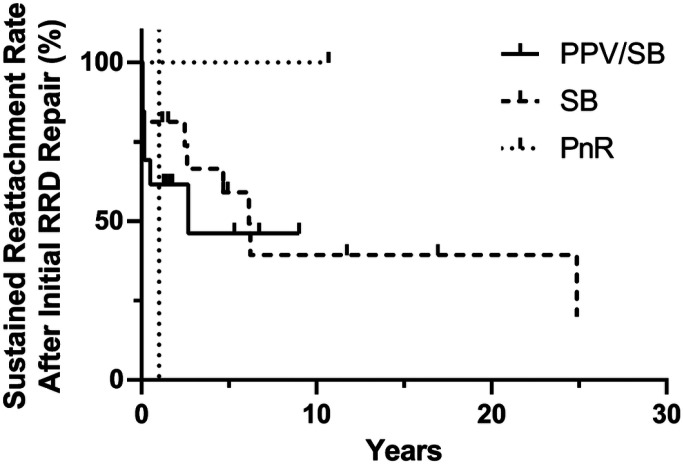
Kaplan-Meier survival curve for sustained retinal reattachment after initial rhegmatogenous retinal detachment repair compared by procedure (p = 0.61, log-rank test). RRD, rhegmatogenous retinal detachment; PPV/SB, combined pars plana vitrectomy and scleral buckling; SB, primary scleral buckling; PnR, pneumatic retinopexy

For initial repair, 13 eyes underwent pars plana vitrectomy combined with scleral buckling (PPV/SB), 16 eyes underwent primary scleral buckling (SB), and 1 eye underwent pneumatic retinopexy (PnR). Recurrent RD occurred in 6 (46%) PPV/SB eyes (5 early and 1 late), 10 (63%) SB eyes (3 early and 7 late), and 0 (0%) PnR eyes (Table 2). The average time to recurrence was 6.8 months (median 1 month) in PPV/SB and 16.2 months (median 14.5 months) in SB. There was no significant difference in survival between the different procedures (Fig. 1, p = 0.61, log-rank test). For those eyes that remained attached, the median follow-up time was 4.8 years (range 1.2–16 years).

We examined potential risk factors for recurrent RD. In terms of features at time of initial repair, PVR (OR 1.34, 95% CI 0.17–13.2) and total RD (OR 1.88, 95% CI 0.41–9.7) trended towards an increased odds of recurrence, while GRT (OR 0.84, 95% CI 0.15–4.6) trended towards a decrease. While none of these factors were statistically significant, they were important in determining the initial surgical procedure. PPV/SB was preferred for eyes presenting with total RD (n = 9, 82%), GRT (n = 8, 100%), and PVR (n = 4, 80%). In terms of surgical procedure, PPV/SB (compared to primary SB) (OR 0.53, 95% CI 0.11–2.4) and 360° formation of chorioretinal adhesion with either laser or cryotherapy (OR 0.47, 95% CI 0.10–2.0) trended towards a decreased odds of recurrence. Correspondingly, 86% of cases with 360° treatment occurred in eyes undergoing PPV/SB.

We next examined characteristics of the eyes at recurrence (Table 3). For the 8 eyes with early recurrent RD, 6 (75%) had PVR leading to recurrence (5 PPV/SB and 1 SB). For the remaining 2 eyes, both of which had undergone primary SB, one had persistent vitreoretinal traction at the pre-existing tear and the other had tears at and anterior to the buckle indentation apex. For those eyes with late recurrent RD, the 1 eye with initial PPV/SB had PVR leading to recurrence. For the other 7 eyes with late recurrence, all had undergone primary SB initially. Four (57%) developed a break posterior to the buckle indentation apex, and the other 3 (43%) had breaks either at or anterior to the buckle indentation apex. For the treatment of recurrent RD in initial SB eyes, 3 eyes (30%, all late recurrence) had barricade laser retinopexy initially performed, but all 10 eyes eventually required vitrectomy. Those eyes with initial PPV/SB had repeat vitrectomy.

**Figure 2:**
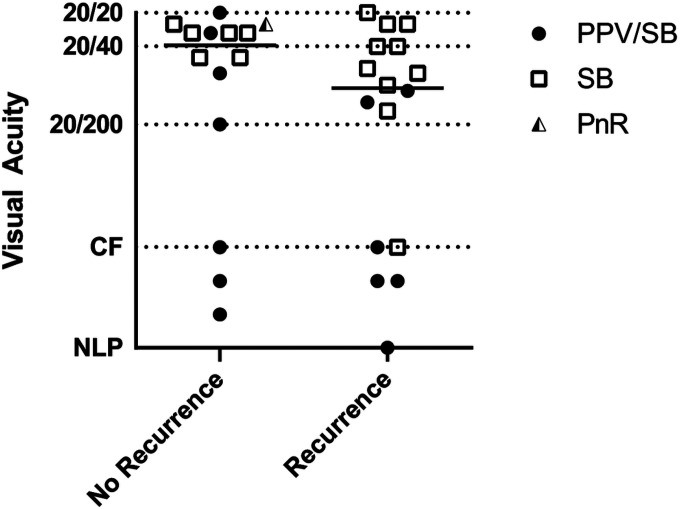
Visual acuity at last follow-up compared by presence of recurrent retinal detachment. Line represents median logMAR value (p = 0.27, Mann Whitney U test). PPV/SB, combined pars plana vitrectomy and scleral buckling; SB, primary scleral buckling; PnR, pneumatic retinopexy

On last documented follow-up, 29 (97%) eyes achieved anatomic reattachment with 3 eyes under silicone oil tamponade. The median LogMAR value on last follow-up was 0.29 (20/40 equivalent) for those eyes without recurrence compared to 0.68 (20/100 equivalent) for those eyes with recurrent RD (Fig. 2, p = 0.27, Mann Whitney U test).

## Discussion

In our study, we found a high rate of recurrent RD (53%) in patients with Stickler Syndrome after initial surgery. In adults, primary rhegmatogenous RD repair is able to achieve a high level of single surgery success (83–92%).^6^ However, pediatric RD are unable to achieve similar levels of single surgery success.^7,8^ Part of this discrepancy may be attributed to the delayed presentation of retinal detachments in the pediatric population. However, another major factor is in the underlying disorder. Rhegmatogenous RD in Stickler Syndrome frequently require multiple surgeries to achieve anatomic success,^4^ and thus, further investigation is necessary to reduce the number of surgeries required for anatomical reattachment and to reduce the risk of late re-detachment. In our population, those eyes with sustained retinal reattachment after initial surgery trended towards improved visual outcomes compared to those eyes with recurrent RD.

On review of the cases, we found no statistically significant difference for recurrence between the initial surgical procedures, although primary SB trended towards an increased odds of recurrent RD compared to combined PPV/SB. Furthermore, when we analyzed the eyes by time to recurrence, we found that most cases of recurrence in PPV/SB occurred soon after surgery while most cases of recurrence after primary SB occurred greater than 1 year after initial surgery. Other studies have found a similar increased average time to recurrent RD with primary SB compared to vitrectomy. Alsharani *et al*. reported on average 6.4 months to recurrence for primary SB compared to 3.04 months for combined PPV/SB, and Abeysiri *et al*. reported 14.1 months for primary SB compared to 4.9 months for all vitrectomy.^9,10^ On the other hand, Taylor *et al*. found the opposite with an average time to recurrence of 34 days with primary SB compared to 156 days for all vitrectomy.^11^ However, their study focused on RD associated with all causes of optically empty vitreous and included non-Stickler Syndrome patients. The high rates of early recurrence in PPV/SB are likely due to the inherent properties of the eyes. Combined PPV/SB is preferred for complex retinal detachments, which skews this treatment selection to eyes at high risk for recurrence. Our cohort exemplifies this where 6 out of 8 early detachments had PVR leading to recurrence with a seventh eye developing another recurrent detachment a few days later due to PVR.

Silicone oil tamponade has been advocated to possibly decrease risk of recurrence.^10^ Eight eyes in our cohort utilized silicone oil tamponade during the initial surgery. Six eyes underwent scheduled oil removal, and 2 of those developed recurrent detachment. One of these eyes was the single case of late recurrence after initial PPV/SB. This eye required oil removal early on for emulsification, but then developed a recurrent RD 2.5 years later with PVR. Overall, only 2 of the initial 8 eyes that utilized silicone oil initially required silicone oil tamponade for anatomic reattachment at last follow-up. This suggests that silicone oil tamponade can be used as a temporizing measure, but we cannot make any de nitive conclusions advocating for its use in initial repair to prevent recurrence.

On the other hand, primary SB is the preferred approach for less complicated retinal detachments. Studies support the use of primary SB for pediatric RD, and it may even be preferred in the pediatric population given their attached posterior hyaloid and clear lens.^7,12^ However, the primary SB cases in our study had a high rate of recurrent RD (63%) with 70% of the recurrences occurring greater than 1 year after initial surgery. Stickler Syndrome is characterized by an abnormal vitreous and vitreoretinal interface, and these factors may play a role in the development of late recurrent RD. When we analyzed primary SB eyes with late recurrence, we saw that 57% of the patients developed a new break posterior to the buckle indentation apex. It is possible that initial vitrectomy may be beneficial to prevent posterior vitreoretinal traction from leading to late recurrent RD, but this requires further study.

One other notable difference in primary SB compared to PPV/SB was in the formation of chorioretinal adhesion. Patients undergoing primary SB typically had focal cryotherapy to the retinal breaks, while patients undergoing combined PPV/SB typically had 360° prophylactic treatment. Studies have demonstrated a decreased incidence of rhegmatogenous RD in Stickler Syndrome eyes that received 360° prophylactic treatment.^3,13,14^ To our knowledge, there have not been any studies assessing the effect of 360° treatment at the time of initial repair in Stickler Syndrome, and this warrants further examination as another means of preventing recurrent RD.

Limitations to our study include its retrospective nature and small patient number. Furthermore, several different surgeons were involved in the RD surgeries, and surgeon preference for various reattachment approaches may have influenced the outcomes. Additionally, different scleral buckle band sizes with different degrees of indentation and placement relative to the vitreous base may have confounded the results from scleral buckle treatment. All of our patients had clinical findings consistent with Stickler Syndrome, but not all of them had molecular confirmation. Furthermore, we had two eyes that experienced recurrent RD greater than 20 years after initial surgery. It is unknown how some of the eyes with seemingly sustained reattachment may fare in the future with longer follow-up.

Overall, we found that early recurrent RD were mostly caused by the development of PVR, while late recurrent RD were mostly due to new retinal breaks, including posterior retinal breaks not supported by the existing scleral buckle indentation. Furthermore, we found that even in seemingly uncomplicated rhegmatogenous RD repair, patients with Stickler Syndrome and long-term sustained retinal reattachment remain at risk for re-detachment and likely require lifelong monitoring. Further studies are needed to explore and identify predictors of late recurrent RD in patients with Stickler Syndrome.

## Figures and Tables

**Table 1:** Features of patients with Stickler Syndrome who underwent rhegmatogenous retinal detachment repair.

Feature	Number (%)
Patients	22
Median age at initial surgery	10 years
Male	16 (73%)
Bilateral RD	9* (41 %)
Median time to fellow eye RD†	3.8 years
Molecular confirmation	14 (64%)
COL2A1	12 (55%)
COL11A1	2 (9%)
COL9A3 - VUS	2 (9%)
Eyes	30
Right Eye	18 (60%)
Recurrent RD	16 (53%)
Median time to recurrent RD	1.49 years

Notes:

* 1 patient with inoperable retinal detachment in fellow eye,

† for patients that did not have bilateral involvement on initial presentation

Abbreviations: *RD*, retinal detachment; *VUS*, variant of uncertain significance

**Table 2:** Comparison of factors at time of initial surgery in eyes that underwent rhegmatogenous retinal detachment repair by those that had a recurrent retinal detachment (early: within 1 year, late: greater than 1 year after initial surgery) and those without recurrence.

Factor	Total	No Recurrence (n = 14, % of total)	Early Recurrence (n = 8, % of total)	Late Recurrence (n = 8, % of total)
Initial Procedure				
PPV/SB	13	7 (50%)	5 (39%)	1 (8%)
Silicone Oil Tamponade	8	4 (50%)	3 (38%)	1 (13%)
SB	16	6 (38%)	3 (19%)	7 (44%)
PnR	1	1 (100%)	-	-
Prior focal laser/cryotherapy	3	2 (67%)	1 (33%)	-
Total RD	11	4 (36%)	6 (55%)	1 (9%)
Type of retinal break				
Single	9	6 (67%)	-	3 (33%)
Multiple	9	3 (33%)	4 (44%)	2 (22%)
GRT	8	4 (50%)	3 (38%)	1 (13%)
N/A*	4	1 (25%)	1 (25%)	2 (50%)
Presence of PVR	5	2 (40%)	3 (60%)	-
Genetic Mutation				
COL2A1	13	8 (62%)	1 (8%)	4 (31%)
COL11A1	4	2 (50%)	1 (25%)	1 (25%)
COL9A3 - VUS	4	2 (50%)	1 (25%)	1 (25%)

Notes:

* retinal break either not found or not mentioned

Abbreviations: *PPV/SB*, combined pars plana vitrectomy and scleral buckling; *SB*, primary scleral buckling; *PnR*, pneumatic retinopexy; *RD*, retinal detachment; *GRT*, giant retinal tear; *PVR*, proliferative vitreoretinopathy; *VUS*, variant of uncertain significance

**Table 3:** Clinical features at time of recurrent retinal detachment compared by time to recurrence

Feature	Early Recurrence (n = 8, %)	Late Recurrence (n = 8, %)
PVR	6 (75%)	1 (12.5%)
Location of retinal break		
At or anterior to buckle apex	2 (25%)	3 (37.5%)
Posterior to buckle apex	1 (12.5%)	4 (50%)
N/A*	5 (62.5%)	1 (12.5%)

Note:

* location of retinal break in relation to buckle apex not mentioned

Abbreviations: *PVR*, proliferative vitreoretinopathy

## Summary

### What was known before:

- Rhegmatogenous retinal detachments in patients with Stickler Syndrome are characterized by recurrent detachments which require multiple surgeries.
- Primary scleral buckling is often utilized for pediatric retinal detachments because of the attached posterior hyaloid and clear lens.

### What this study adds:

- In Stickler Syndrome, early recurrent retinal detachments are mostly due to proliferative vitreoretinopathy, while late recurrent retinal detachments are mostly due to new retinal breaks.
- Patients with Stickler Syndrome and seemingly sustained retinal reattachment remain at risk for late recurrent retinal detachment.
